# Patient-Specific Computer Simulation to Predict Conduction Disturbance With Current-Generation Self-Expanding Transcatheter Heart Valves

**DOI:** 10.1016/j.shj.2022.100010

**Published:** 2022-03-29

**Authors:** Cameron Dowling, Robert Gooley, Liam McCormick, Hashrul N. Rashid, James Dargan, Faisal Khan, Sami Firoozi, Stephen J. Brecker

**Affiliations:** a MonashHeart, Monash Health and Monash Cardiovascular Research Centre, Monash University, Melbourne, Australia; b Cardiovascular Clinical Academic Group, St. George’s, University of London and St. George’s University Hospitals NHS Foundation Trust, London, UK

**Keywords:** Aortic valve stenosis, Computer simulation, Finite element analysis, Heart valve prosthesis implantation, Transcatheter aortic valve replacement

## Abstract

**Background:**

Patient-specific computer simulation may predict the development of conduction disturbance following transcatheter aortic valve replacement (TAVR). Validation of the computer simulations with current-generation devices has not been undertaken.

**Methods:**

A retrospective study was performed on patients who had undergone TAVR with a current-generation self-expanding transcatheter heart valve (THV). Preprocedural computed tomography imaging was used to create finite element models of the aortic root. Procedural contrast angiography was reviewed, and finite element analysis performed using a matching THV device size and implantation depth. A region of interest corresponding to the atrioventricular bundle and proximal left bundle branch was identified. The percentage of this area (contact pressure index [CPI]) and maximum contact pressure (CPMax) exerted by THV were recorded. Postprocedural electrocardiograms were reviewed, and major conduction disturbance was defined as the development of persistent left bundle branch block or high-degree atrioventricular block.

**Results:**

A total of 80 patients were included in the study. THVs were 23- to 29-mm Evolut PRO (n = 53) and 34-mm Evolut R (n = 27). Major conduction disturbance occurred in 27 patients (33.8%). CPI (28.3 ± 15.8 vs. 15.6 ± 11.2%; *p* < 0.001) and CPMax (0.51 ± 0.20 vs. 0.36 ± 0.24 MPa; *p* = 0.008) were higher in patients who developed major conduction disturbance. CPI (area under the receiver operating characteristic curve [AUC], 0.74; 95% CI, 0.63-0.86; *p* < 0.001) and CPMax (AUC, 0.69; 95% CI, 0.57-0.81; *p* = 0.006) demonstrated a discriminatory power to predict the development of major conduction disturbance.

**Conclusions:**

Patient-specific computer simulation may identify patients at risk for conduction disturbance after TAVR with current-generation self-expanding THVs.

## Introduction

Transcatheter aortic valve replacement (TAVR) is associated with improved clinical outcomes, when compared to surgery,^1^ and has now become the dominant therapy for treating patients with severe aortic stenosis.^2^ However, TAVR is associated with a higher incidence of left bundle branch block (LBBB)^3^ and permanent pacemaker (PPM) implantation,^4^^,^^5^ than surgery, and patients who develop these complications are at a higher risk for adverse clinical outcomes.^6^

As TAVR continues to expand into younger, lower-risk cohorts, identifying patients who may be at risk for conduction disturbance and developing strategies to minimize its occurrence is important. One potential solution to this challenge is patient-specific computer simulation. The technology uses a patient’s preprocedural cardiac computed tomography (CT) scan to create a computer model of the aortic root. A region of interest corresponding to the atrioventricular bundle is identified, and conduction disturbance modeling is performed. The computer simulations have been validated in patients treated with early-generation self-expanding transcatheter heart valves (THVs)^7^, ^8^, ^9^ but have not been studied in patients treated with current-generation devices, which, due to changes in either their THV frame design^10^ or the addition of a pericardial wrap,^11^ may display different interactions with the conduction system.

In this study, we wished to validate the conduction disturbance modeling in patients treated with current-generation self-expanding THVs and we hypothesized that patient-specific computer simulation could predict the development of conduction disturbance. Furthermore, we sought to examine whether computer simulation could identify patients at risk for prolonged hospitalization and long-term adverse clinical outcomes and we hypothesized that the patient-specific computer simulations would also be predictive of these clinical outcomes.

## Methods

A retrospective study was performed on all patients who had undergone TAVR with a current-generation 23- to 29-mm Evolut PRO or 34-mm Evolut R (Medtronic, Minneapolis, MN) self-expanding THV across 2 study sites (Monash Medical Centre, Melbourne, Australia and St. George’s Hospital, London, United Kingdom). Patients were included if their preprocedural cardiac CT imaging had adequate right-sided contrast opacification to visualize the membranous septum and if procedural contrast angiography had adequate aortic root opacification to visualize the THV implantation depth. Patients were excluded if they had pre-existing LBBB or PPM implantation.

### Patient Characteristics

Patient characteristics were obtained from the local electronic databases. National electronic records were reviewed to ascertain mortality status.

### Cardiac CT Analysis

Cardiac CT imaging was acquired using either an Aquilon ONE (Canon Medical Systems Corporation, Otawara, Japan) 320-slice or SOMATOM Definition Flash (Siemens Healthcare, Erlangen, Germany) 128-slice scanner, with 0.5-mm slice thickness. Imaging was used to create aortic valve perpendicular plane and 3-dimensional reconstructions with 3mensio Structural Heart, version 9.1 (Pie Medical Imaging, Maastricht, the Netherlands). Aortic valves were classified using the Sievers^12^ and TAVR-directed bicuspid aortic valve imaging (BAVi)^13^ systems. Aortic root dimensions^14^ were measured. Calcium volume analysis was performed in the upper leaflet, device landing zone and left ventricular outflow tract.^15^ Further calcium volume analysis was performed in these regions in the noncoronary, right and left leaflet segments.^16^

### Computer Simulation

**Figure 1:**
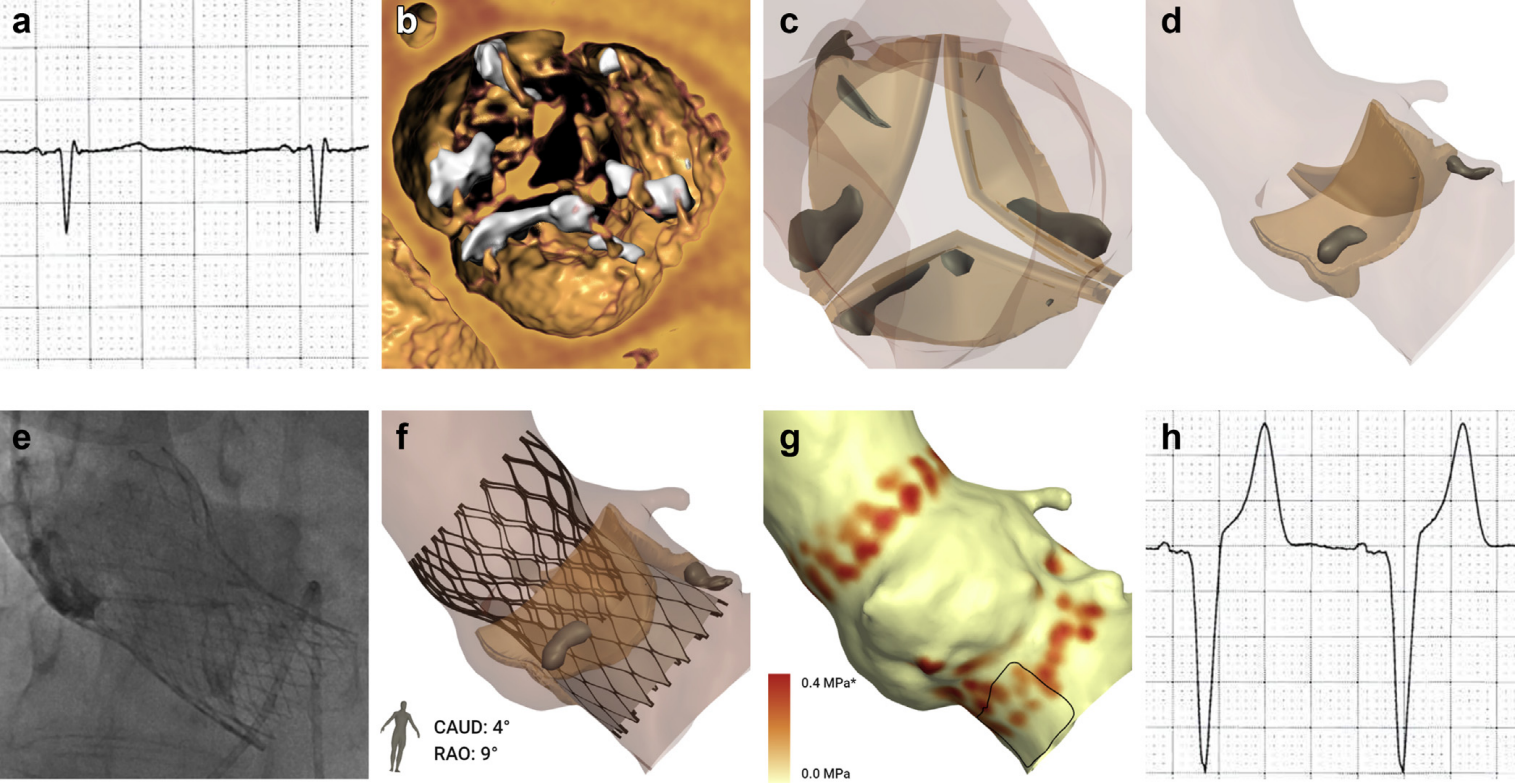
**Patient-specific computer simulation.** (a) Baseline ECG demonstrated no significant conduction disturbance. (b) Cardiac CT imaging demonstrated a trileaflet valve with moderate valvular calcification. (c and d) A finite element model of the aortic root was created. (e) Procedural contrast angiography was reviewed and (f) finite element analysis performed to simulate the same implantation depth. (g) The membranous septum was identified in 3 locations, and a region of interest defined corresponding to the atrioventricular bundle and proximal left bundle branch. The percentage of this area (contacted pressure index [CPI]) and maximum pressure exerted by the THV (CPMax) was recorded. In this example, computer simulation predicts major conduction disturbance (CPI 51%, CPMax 0.50 MPa). (h) Postprocedural ECGs demonstrated left bundle branch block. ∗Values above this maximum are displayed in the high color in the scale. Abbreviations: CT, computed tomography; ECG, electrocardiogram; THV, transcatheter heart valve.

Patient-specific computer simulation was performed using FEops HEARTguide technology (FEops nv, Ghent, Belgium) using previously described methods (Figure 1).^8^ In brief, finite element models of the aortic root were created from preprocedural cardiac CT imaging, with the aortic leaflets (E = 0.6 MPa, ν = 0.3), wall (E = 2 MPa, ν = 0.45), and calcium (E = 4 MPa, ν = 0.3, Yield stress = 0.6 MPa) being modeled with differing mechanical properties.^17^ To account for the impact of the surrounding cardiac structures, spring elements were added at each node of the aortic wall. In the finite element analysis simulation, the THV was first crimped into the sheath and positioned coaxially within the aortic root. A force was then applied to displace the device toward the outer curvature of the aorta, and the sheath was then retracted, leading to the expansion of the THV.

Procedural contrast angiography was reviewed and finite element analysis simulation performed to simulate the same implantation depth of the THV in the corresponding projection angle. The depth of implantation was measured in the computer model at the noncoronary and left coronary cusps.

### Conduction Disturbance Modeling

The force exerted by the THV on the patient’s anatomy was extracted at the end of deployment, as direct output of the finite element analysis simulation. This nodal force was then translated into pressure per element, taking into account the size of each aortic root model’s element in the region where the membranous septum is located. The membranous septum was located in 3 locations (noncoronary cusp, mid-course, and right coronary cusp) and was extended 25° laterally from the right coronary cusp. A region of interest was defined by extending 15 mm caudal to the aortic annulus (Figure 1g). This region is an anatomical surrogate for the atrioventricular bundle and proximal left bundle branch.^18^ Two measures of conduction disturbance were recorded. The first was contact pressure index (CPI), which was defined as the percentage of the region of interest subject to pressure by the THV. The second was the maximum contact pressure (CPMax) exerted by the THV on the region of interest. A mesh sensitivity analysis on the aortic root was performed to ensure that the recorded pressure-related outputs were mesh independent.

### Procedural Characteristics

TAVR procedural reports were reviewed, and characteristics recorded. Procedural contrast angiography was reviewed and used to guide depth of implantation in the computer model, as previously described.

### Conduction Disturbance Analysis

Preoperative and postoperative electrocardiograms up to 72 hours postprocedure were reviewed, and conduction disturbances recorded. Major conduction disturbance was defined as the development of a persistent LBBB or the development of persistent high-degree atrioventricular block (second-degree atrioventricular block Mobitz type II or third-degree atrioventricular block). In-hospital PPM implantation procedures and indications were recorded.

### Statistical Analysis

Statistical analysis was performed using SPSS, version 28.0 (IBM Corporation, Armonk, New York). Continuous variables are presented as mean ± standard deviation, and categorical variables as frequencies (percentage). The means of groups were compared with a 2-tailed Student’s t-test, with a *p* value < 0.05 considered statistically significant. The means of more than 2 groups were compared with analysis of variance. The median of 2 groups was compared with a Mann-Whitney test. Contingency analysis on 2 groups was performed using a Fisher’s exact test and on more than 2 groups with a chi-squared test. Discriminatory power was tested using the area under the receiver operating characteristic curve (AUC). Potential predictors for clinical outcomes were assessed using univariate binary logistic regression analysis, with a predictor with a *p* value < 0.1 then included in the multivariate analysis. Time-to-event analysis was performed with the use of Kaplan-Meier estimates and Cox regression and was compared with the use of the log-rank test.

## Results

Between March 2017 and February 2021, a total of 225 patients underwent implantation with a current-generation self-expanding THV. Of these patients, 71 patients (31.6%) had pre-existing LBBB or PPM, leaving a total of 154 for potential inclusion in the study. However, 74 of these patients (48.1%) did not have adequate CT imaging quality (inadequate right-sided contrast opacification in 67 patients, motion artefact in 6 patients, and a very narrow left ventricular outflow tract in 1 patient), leaving a total of 80 patients for inclusion in the study.

### Baseline Characteristics

Baseline patient characteristics demonstrated an elderly patient cohort (age, 79.8 ± 10.8 years) at increased risk for surgery (European System for Cardiac Operative Risk Evaluation II, 5.0 ± 4.2%) (Table 1). There was a high prevalence of previous atrial fibrillation or atrial flutter (35.0%).

**Table 1:** Baseline patient, cardiac computed tomography, and electrocardiographic characteristics

Characteristic	n = 80
Age, yrs	79.8 ± 10.8
Male	49 (61.3)
EuroSCORE II (%)	5.0 ± 4.2
Aortic valve morphology	
Bicuspid	4 (5.0)
Sievers classification	
Sievers type 0	3 (3.8)
Sievers type 1	1 (1.3)
TAVR-directed BAVi morphological classification	
Tricommissural	1 (1.3)
Bicommissural raphe type	2 (2.5)
Bicommissural non-raphe type	1 (1.3)
Tricuspid	76 (95.0)
Aortic root dimensions	
Left ventricular outflow tract diameter∗ (mm)	25.4 ± 3.1
Aortic annulus diameter∗ (mm)	25.1 ± 2.5
Sinus of Valsalva diameter∗ (mm)	34.3 ± 4.0
Sinotubular junction diameter∗ (mm)	28.9 ± 3.5
Ascending aorta diameter∗ (mm)	32.1 ± 3.2
Aortic leaflet calcium volume (mm^3^)	343.8 ± 344.1
Noncoronary leaflet	172.1 ± 198.4
Right coronary leaflet	80.2 ± 95.4
Left coronary leaflet	91.5 ± 99.7
Upper leaflet calcium volume (mm^3^)	325.7 ± 329.4
Noncoronary leaflet	164.1 ± 188.8
Right coronary leaflet	78.2 ± 93.3
Left coronary leaflet	82.8 ± 91.0
Device landing zone calcium volume (mm^3^)	24.9 ± 37.9
Noncoronary leaflet	10.0 ± 25.3
Right coronary leaflet	1.7 ± 4.9
Left coronary leaflet	11.8 ± 23.3
Left ventricular outflow tract calcium volume (mm^3^)	16.8 ± 33.1
Noncoronary leaflet	4.2 ± 14.7
Right coronary leaflet	0.1 ± 1.1
Left coronary leaflet	12.5 ± 29.6
Membranous septum depth (mm)	3.4 ± 2.2
Noncoronary cusp	5.1 ± 3.0
Mid-course	3.5 ± 2.3
Right coronary cusp	1.7 ± 2.2
Cardiac rhythm	
Sinus rhythm	56 (70.0)
Atrial fibrillation	22 (27.5)
Atrial flutter	2 (2.5)
Conduction abnormalities	
Right bundle branch block	13 (16.3)
First-degree atrioventricular block	15 (18.8)

BAVi = bicuspid aortic valve imaging, EuroSCORE II = European System for Cardiac Operative Risk Evaluation II, TAVR = transcatheter aortic valve replacement.

∗ Perimeter-derived values.

### Cardiac CT Analysis

The majority of patients had tricuspid aortic valve morphology (95.0%) (Table 1). Aortic root dimensions^14^ and calcium volumes (including total and segmental volumes)^15^^,^^16^ were similar to published reference values. The device landing zone calcium volume at the noncoronary leaflet was 10.0 ± 25.3 mm^3^. The mean membranous septum depth was 3.4 ± 3.2 mm.

### Baseline Electrocardiographic Characteristics

Baseline electrocardiogram characteristics demonstrated a high prevalence of atrial fibrillation or flutter (30.0%), and several patients had pre-existing right bundle branch block (16.3%) (Table 1).

### Procedural Characteristics

Procedural characteristics demonstrated that the majority of cases were performed under local anesthesia with sedation (81.3%) (Supplemental Table 1). Predilation was frequently performed (48.8%). The most commonly implanted THVs were the 29-mm Evolut PRO (43.8%) and 34-mm Evolut R (33.8%). The mean implantation depth was 6.2 ± 2.3 mm.

### Conduction Abnormalities

**Figure 2:**
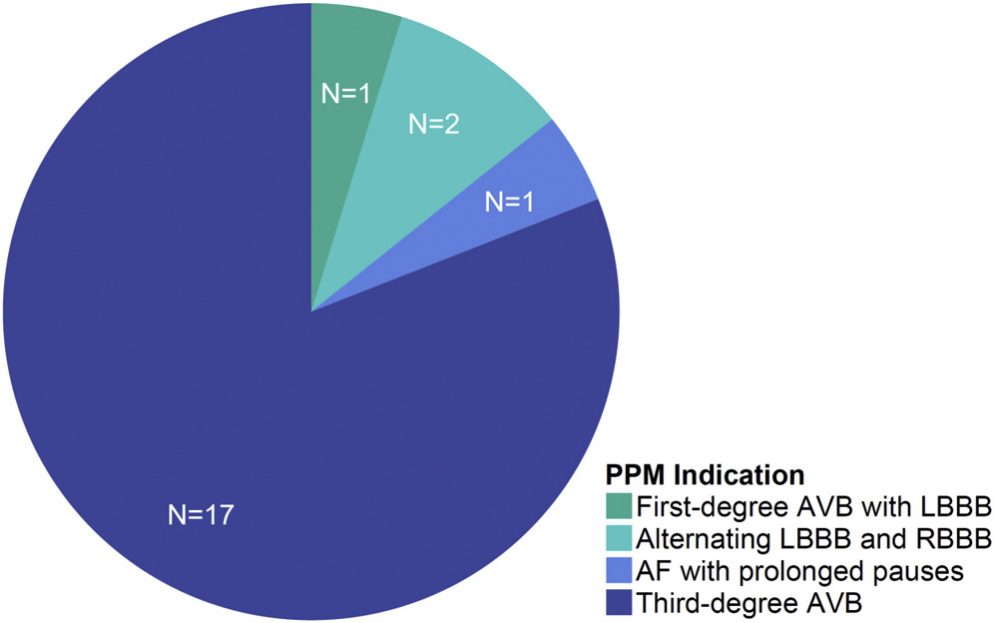
**Indications for permanent pacemaker implantation.** The most common indication for PPM was third-degree atrioventricular block. Abbreviations: AF, atrial fibrillation; AVB, atrioventricular block; LBBB, left bundle branch block; PPM, permanent pacemaker; RBBB, right bundle branch block.

New conduction abnormalities developed in 46 patients (57.5%) (Table 2). Major conduction disturbance developed in 27 patients (33.8%), including 24 patients who developed persistent LBBB (30.0%) and 15 patients (18.8%) who developed persistent high-degree atrioventricular block. There were 21 patients (26.3%) who underwent implantation of a PPM, and the most common indication for a PPM was for transient or persistent third-degree atrioventricular block (81.0%) (Figure 2).

**Table 2:** Conduction abnormalities

Outcome	n = 80
New conduction abnormalities	
Left bundle branch block	24 (30.0)
Right bundle branch block	2 (2.5)
First-degree atrioventricular block	4 (5.0)
Second-degree atrioventricular block	0 (0.0)
Third-degree atrioventricular block	12 (15.0)
Permanent pacemaker implantation	21 (26.3)

### Computer Simulation

The mean case processing time was 106 ± 27 minutes. The mean CPI was 19.9 ± 14.2%. CPI was similar across all 4 THV prostheses (*p* = 0.11). The mean CPMax was 0.41 ± 0.24 MPa, and CPMax was also similar across all 4 devices (*p* = 0.13).

### Predictors of Major Conduction Disturbance

**Figure 3:**
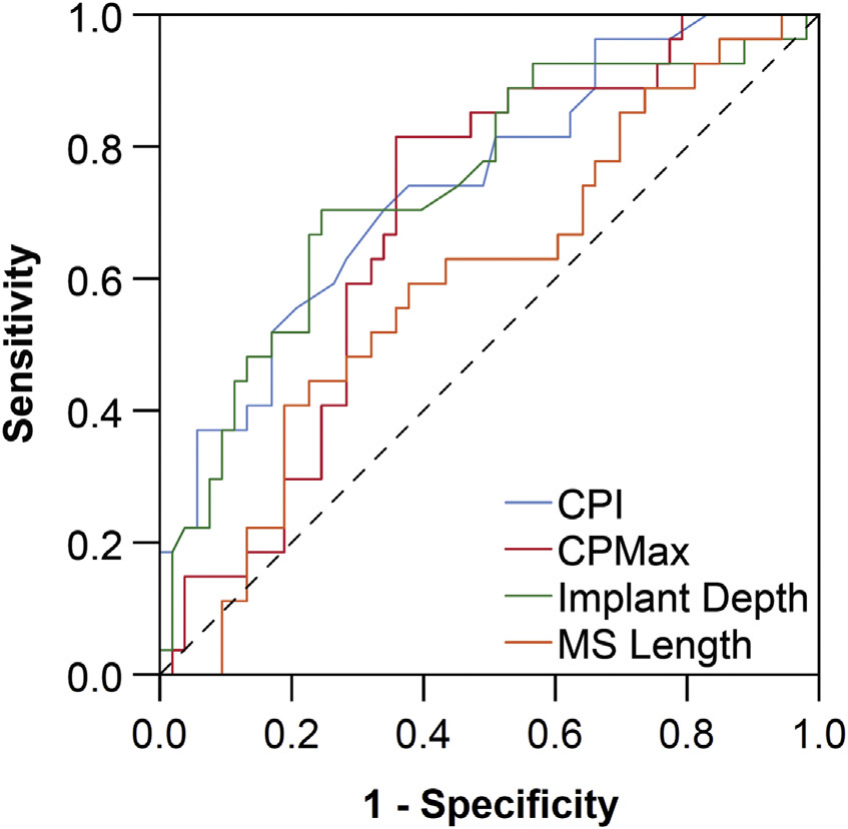
**Discriminatory power to predict the development of major conduction abnormalities.** Contact pressure index (CPI), maximum contact pressure (CPMax), and THV implantation depth were predictive of major conduction abnormalities, whereas membranous septum (MS) length did not demonstrate any discriminatory power. Diagonal segments are produced by times. Abbreviation: THV, transcatheter heart valve.

The mean CPI was 20 ± 14%. CPI was higher in patients who developed major conduction disturbance (28.3 ± 15.8 vs. 15.6 ± 11.2%; *p* < 0.001). CPI demonstrated a discriminatory power to predict major conduction disturbance (AUC, 0.74; 95% confidence interval [CI], 0.63-0.86; *p* < 0.001) (Figure 3). The optimal cutoff for predicting major conduction disturbance was a CPI ≥20%, representing a sensitivity of 70%, specificity of 66%, positive predictive value (PPV) of 51%, negative predictive value (NPV) of 81%, and accuracy 67.5%. There was no association between THV prosthesis size and CPI (*p* = 0.11 for linear trend).

The mean CPMax was 0.41 ± 0.24 MPa. CPMax was higher in patients who developed major conduction disturbance (0.51 ± 0.20 vs. 0.36 ± 0.24 MPa; *p* = 0.008). CPMax demonstrated a discriminatory power to predict major conduction disturbance (AUC, 0.69; 95% CI, 0.57-0.81; *p* = 0.006). The optimal cutoff for predicting major conduction disturbance was a CPMax of 0.40 MPa, representing a sensitivity of 22%, specificity of 96%, PPV of 75%, NPV of 71%, and accuracy of 71.3%. Increasing THV prosthesis size was associated with a higher CPMax (*p* = 0.02 for linear trend).

The incidence of major conduction disturbance was higher in patients with a CPI ≥20% (51.3% vs. 18.6%; *p* = 0.004) and in patients with a CPMax ≥0.40 MPa (53.7% vs. 12.8%; *p* < 0.001). Furthermore, major conduction disturbance was particularly high in patients with both of these factors (62.1%), when compared with patients who had only one (25.0%) or none (12.9%) of these factors present on computer simulation (*p* = 0.002).

Established risk factors for major conduction disturbance were predictive of this complication, including implantation depth (AUC, 0.74; 95% CI, 0.62-0.86; *p* < 0.001). However, membranous septum length did not demonstrate a discriminatory power to predict major conduction disturbance (AUC, 0.60; 95% CI, 0.47-0.73; *p* = 0.16).

Leaflet calcium volume (AUC, 0.47; 95% CI, 0.34-0.61; *p* = 0.47), upper leaflet calcium volume (AUC, 0.78; 95% CI, 0.35-0.62; *p* = 0.79), device landing zone calcium (AUC, 0.49; 95% CI, 0.35-0.63; *p* = 0.87) and left ventricular outflow tract calcium (AUC, 0.54; 95% CI, 0.41-0.68; *p* = 0.54) were not predictive for major conduction disturbance. Furthermore, for each of these locations, calcium volumes at the noncoronary cusp, right coronary cusp, and left coronary cusp were not predictive of major conduction disturbance, including device land zone calcium at the noncoronary cusp (AUC, 0.47; 95% CI, 0.33-0.61; *p* = 0.69).

In a univariate analysis, a CPI ≥20%, a CPMax ≥0.40 MPa, and an implantation depth ≥5 mm were predictors for major conduction disturbance (Table 3). On multivariate analysis, only a CPMax 0.40 MPa remained an independent predictor for major conduction disturbance.

**Table 3:** Predictors of major conduction disturbance

Predictor	Univariate analysis		Multivariate analysis	
	Odds ratio (95% CI)	*p* Value	Odds ratio (95% CI)	*p* Value
CPI ≥20%	4.62 (1.69-12.60)	0.003	1.72 (0.51-5.80)	0.38
CPMax ≥0.40 MPa	7.87 (2.57-24.17)	<0.001	5.23 (1.51-18.08)	0.009
Implantation depth ≥5 mm	5.90 (1.25-27.85)	0.03	3.10 (0.56-17.16)	0.20
Membranous septum length ≤5 mm	2.49 (0.74-8.36)	0.14		
Implantation depth > membranous septum length	1.87 (0.55-6.41)	0.32		
Pre-existing RBBB	2.74 (0.82-9.19)	0.10		
34-mm Evolut R THV	2.03 (0.77-5.33)	0.15		

CI = confidence interval, CPI = contact pressure index, CPMax = maximum contact pressure, NCC = noncoronary cusp, RBBB = right bundle branch block, THV = transcatheter heart valve.

### Predictors of PPM Implantation

The incidence of PPM implantation was higher in patients who had a CPI ≥20% than in patients with a CPI <20% (40.5% vs. 14.0%; *p* = 0.01). Frequency of PPM implantation was also higher in patients with a CPMax ≥0.40 MPa than in patients with a CPMax <0.40 MPa (39.0% vs. 12.8%; *p* = 0.01). Furthermore, the incidence of PPM implantation was the highest in patients who had both of these factors (61.9%) when compared to patients who had only one (23.8%) or none of these factors (14.3%) (*p* = 0.008).

### Patient-Specific THV Positioning

**Figure 4:**
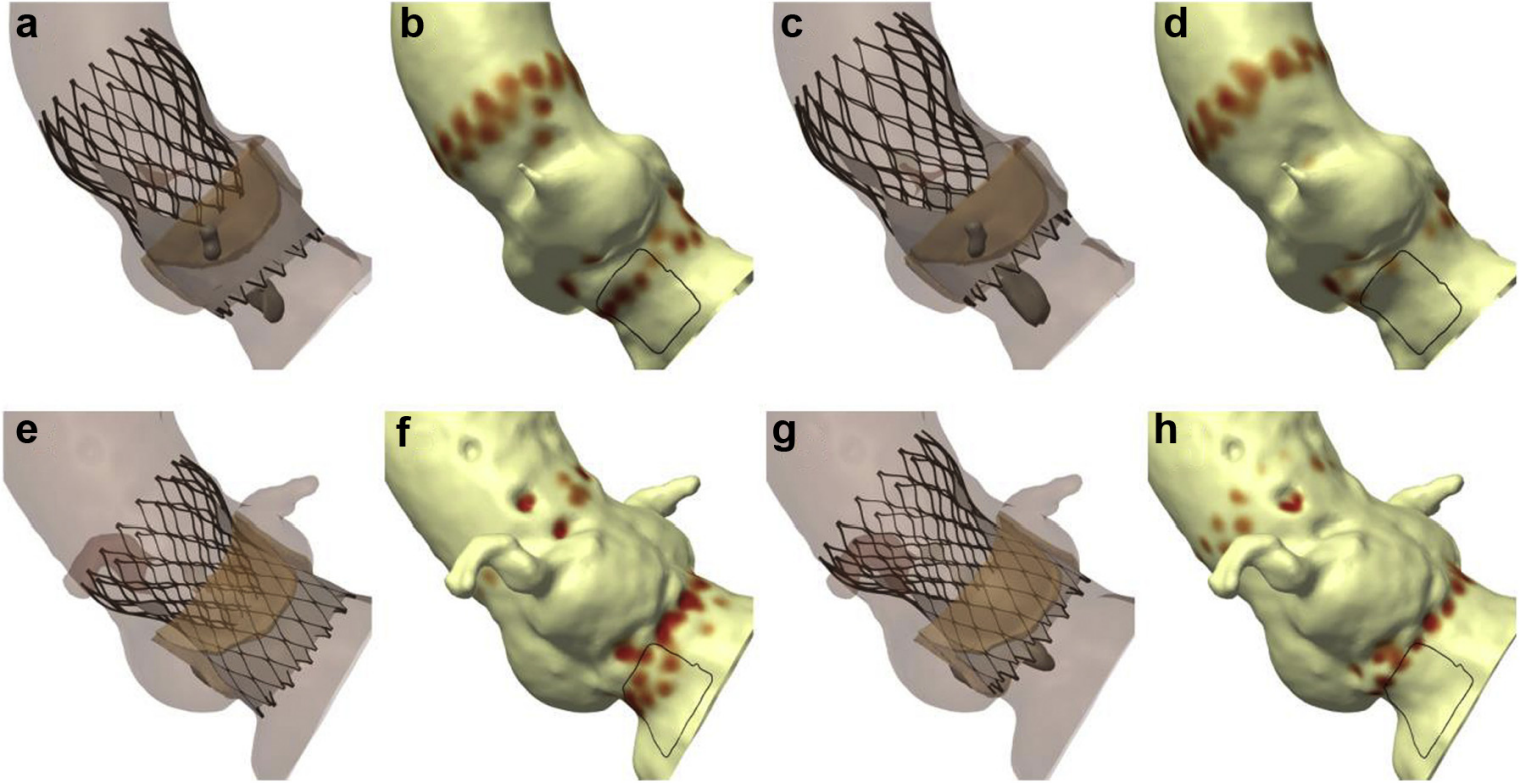
**Patient-specific THV positioning.** (a) A patient underwent TAVI with a 26-mm Evolut PRO THV, developing LBBB. (b) Conduction disturbance simulation predicts major conduction disturbance (CPI 25%, CPMax 0.82 MPa). (c and d) Computer simulation suggests that implanting the THV in a high position will reduce the risk of conduction disturbance (CPI 6%, CPMax 0.11 MPa). (e) A patient underwent TAVI with a 34-mm Evolut R THV, requiring implantation of a PPM for third-degree atrioventricular block. (f) Conduction disturbance modeling predicts major conduction disturbance (CPI 48%, CPMax 0.46 MPa). (g and h) Computer simulation of a high implant suggests that the patient may remain at risk major conduction disturbance (CPI 6%, CPMax 0.51 MPa). Abbreviations: CPI, contact pressure index; CPMax, maximum contact pressure; LBBB, left bundle branch block; PPM, permanent pacemaker; THV, transcatheter heart valve.

Of the 27 patients who developed major conduction disturbance, computer simulation correctly predicted this clinical outcome (CPI ≥20% and/or CPMax ≥0.40 MPa) in 23 patients (85.2%). In those patients, additional computer simulations were performed targeting a high implantation depth (0-3 mm). When compared to the simulation that matched the implanted THV, these additional simulations had a higher implantation depth (2.1 ± 1.2 vs. 7.9 ± 2.6 mm; *p* < 0.001), a lower CPI (4 ± 4 vs. 32 ± 15%; *p* < 0.001), and a lower CPMax (0.22 ± 0.22 vs. 0.56 ± 0.18 MPa; *p* < 0.001). Computer simulations suggested that even with a high implantation depth, 5 of these patients (21.7%) were predicted to have major conduction disturbance (CPMax ≥0.40 MPa) (Figure 4).

### Length of Stay

**Figure 5:**
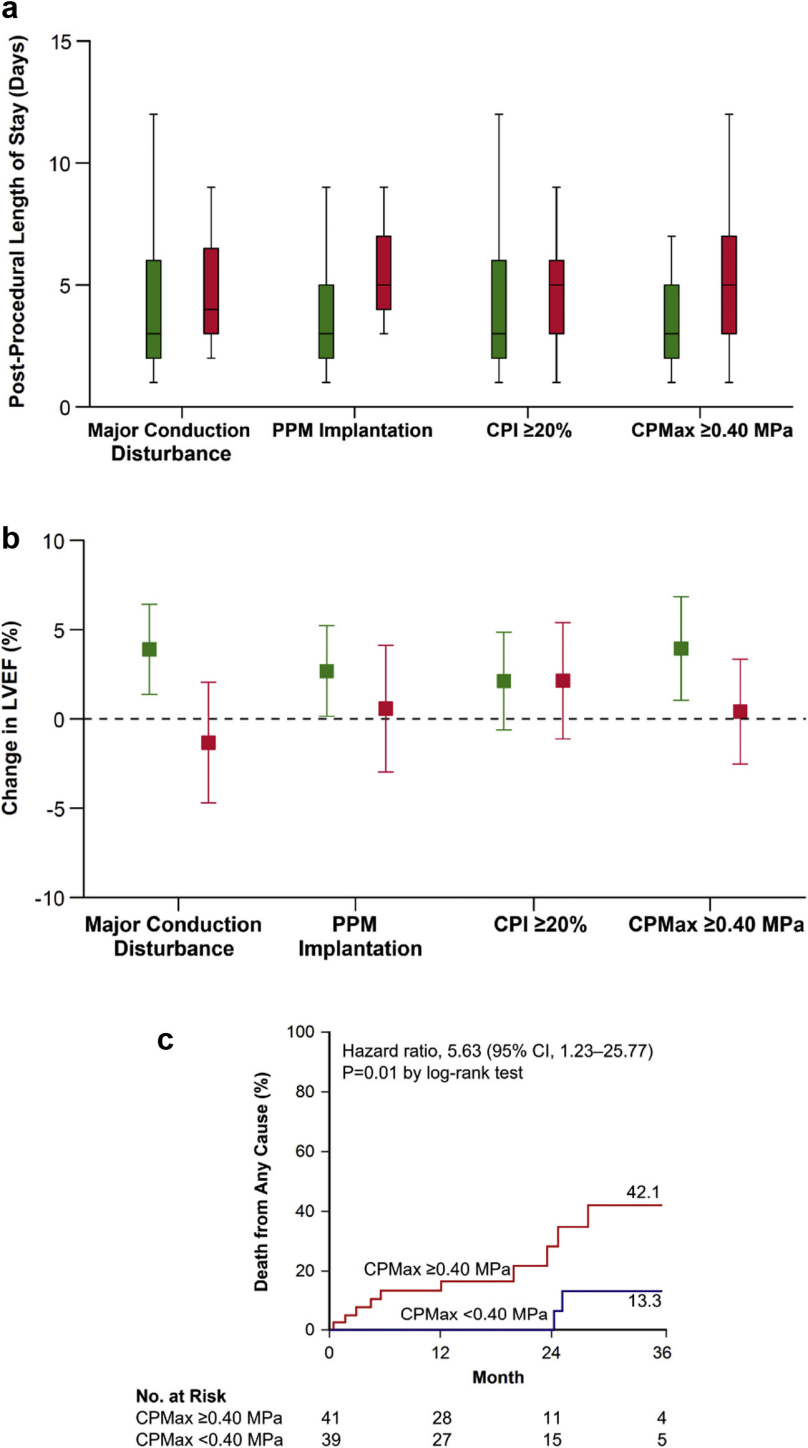
**Patient clinical outcomes.** (a) Postprocedural length of stay was longer in patients who required implantation of a PPM and in patients where computer simulation predicted a higher contact pressure from the THV. (b) LVEF increased in patients who did not develop major conduction disturbance, in patients who did not have a PPM implantation, and in patients where computer simulation predicted a low maximum contact pressure. (c) At 3 years, an elevated CPMax was associated with a higher risk of death from any cause. Green boxplots/bars represent the absence of factors, and red boxplots represent the presence of factors. Error bars represent 95% CI. Abbreviations: CI, confidence interval; CPMax, maximum contact pressure; LVEF, left ventricular ejection fraction; PPM, permanent pacemaker; THV, transcatheter heart valve.

Median postprocedural length of stay was 4.0 days (interquartile range, 2.0-6.0 days). Median postprocedural length of stay was similar in patients who developed major conduction disturbance, when compared to patients who did not develop major conduction disturbance (3.0 vs. 4.0 days; *p* = 0.07) (Figure 5a). Median postprocedural length of stay was longer in patients who required PPM implantation (3.0 vs. 5.0 days; *p* = 0.003).

There was no significant different in median postprocedural length of stay for patients with a CPI ≥20% (3.0 vs. 5.0 days; *p* = 0.08) but it was longer for patients with a CPMax ≥0.40 MPa (3.0 vs. 5.0 days; *p* = 0.02).

### Echocardiographic Outcomes

At 30 days, there was an increase in left ventricular ejection fraction (LVEF) (mean difference, 2.2%; 95% CI, 0.2-4.3%; *p* = 0.03). Patients who did not develop major conduction disturbance had an increase in LVEF (mean difference, 3.8%; 95% CI, 1.3-6.3%; *p* = 0.004), whereas patients who developed major conduction disturbance did not have any significant change in LVEF (mean difference, −1.3%; 95% CI, −4.7 to 2.0%; *p* = 0.42) (Figure 5b). Patients who did not require PPM implantation had an increase in LVEF (mean difference, 2.7%; 95% CI, 0.1-5.2%; *p* = 0.04), whereas patients who required a PPM implantation did not have any significant change in LVEF (mean difference, 0.3%; 95% CI, −3.2 to 3.8%; *p* = 0.85).

Patients with both a CPI <20% (mean difference, 2.0%; 95% CI, −0.7 to 4.7%; *p* = 0.15) and a CPI ≥20% (mean difference, 2.1%; 95% CI, −1.1 to 5.4; *p* = 0.20) had no significant change in LVEF. Patients with a CPMax <0.40 MPa had an increase in LVEF (mean difference, 3.8%; 95% CI, 0.9-6.7%; *p* = 0.01), whereas patients with a CPMax ≥0.40 MPa did not have any significant change in LVEF (mean difference, 0.4%; 95% CI, −2.5 to 3.3%; *p* = 0.78).

### Long-Term Outcomes

At 3 years, patients with major conduction disturbance did not have a significantly higher risk of death from any cause than patients who did not develop this clinical outcome (36.1 vs. 24.1%; hazard ratio, 2.06; 95% CI, 0.66-6.39; *p* = 0.20 by log-rank test). Patients requiring a PPM did not have a significantly higher risk of death than other patients (41.3% vs. 23.6%; hazard ratio, 2.41; 95% CI, 0.76-7.65; *p* = 0.12 by log-rank test); however, patients requiring PPM for third-degree atrioventricular block were at a higher risk for death from any cause than other patients (50.3% vs. 22.4%; hazard ratio, 3.33; 95% CI, 1.05-10.57; *p* = 0.03 by log-rank test).

Patients with a CPI ≥20% did not have a significantly higher risk of death than patients with a CPI <20% (33.0% vs. 23.2%; hazard ratio, 1.75; 95% CI, 0.55-5.52; *p* = 0.34 by log-rank test). Patients with a CPMax ≥40.0 MPa had a higher risk of death than patients with a CPMax <40.0 MPa (42.1% vs. 13.3%; hazard ratio, 5.63; 95% CI, 1.23-25.77; *p* = 0.01 by log-rank test) (Figure 5c).

## Discussion

In this study, we investigated whether patient-specific computer simulation might predict the development of major conduction disturbance after TAVR with current-generation THVs. We demonstrated that computer simulation may predict the development of significant conduction disturbance. Furthermore, there were exploratory suggestions that computer modeling might also predict a number of important associated adverse clinical outcomes, including PPM implantation, postprocedural length of stay, lack of improvement in LVEF, and long-term mortality.

Conduction disturbance after TAVR is an important consideration as TAVR increasingly enters younger, lower-risk patient cohorts. LBBB may be associated with a number of adverse clinical outcomes, including a higher incidence of PPM,^19^ lack of improvement in LVEF,^19^ poorer functional status,^19^ rehospitalization for heart failure,^6^ all-cause mortality,^6^^,^^20^ and sudden cardiac death,^21^ although it should be noted that there exists conflicting evidence surrounding the prognostic importance of LBBB.^19^^,^^22^^,^^23^ Furthermore, PPM implantation has been associated with a number of adverse clinical outcomes, including a lack of improvement in LVEF, heart failure rehospitalization, and mortality;^6^^,^^24^, ^25^, ^26^ however, again there are conflicting data surrounding the association of PPM with long-term mortality.^24^^,^^27^

With this as a background, techniques to identify patients at risk for conduction disturbance and minimize the risk of this procedural outcome would be desirable. One potential solution is patient-specific computer simulation, a technology that has been validated in patients treated with early-generation self-expanding THVs.^8^ In that study, computer simulation was able to identify patients at risk for major conduction disturbance, and the optimal thresholds for predicting conduction disturbance were a CPI ≥14% and a CPMax ≥0.39 MPa.

In this study, we confirmed that the computer simulations could predict major conduction disturbance with acceptable diagnostic performance when simulating current-generation devices. Interestingly, the optimal threshold of CPI to predict major conduction disturbance was slightly higher in our study (CPI ≥20%) than in previous work, and this might be attributable to differences in the design of the current-generation Evolut PRO THV, which has an outer pericardial wrap that might potentially minimize conduction disturbance, as has been suggested by real-world observation data.^28^ Furthermore, mean CPI in this study (20%) was lower than in previous studies, possibly reflecting the ability for operators to achieve a higher implantation depth with recapturable devices.^29^ The optimal CPMax for predicting major conduction disturbance (CPMax ≥0.40 MPa) was similar in this study, confirming the important role that this variable plays in predicting conduction disturbance. Indeed, on a multivariate analysis, CPMax remained an independent predictor of major conduction disturbance.

Identifying patients at risk for PPM implantation is particularly important for young, low-risk patients, and in this study, we demonstrated that computer simulation could also identify patients at risk for PPM implantation and that this risk was highest for patients who had both a CPI ≥20% and a CPMax ≥0.40 MPa. A number of other factors have been attributed to the risk of PPM implantation, including implantation depth^10^^,^^11^ and membranous septum length.^30^ We confirmed that implantation depth was predictive of major conduction disturbance; however, we did find neither membranous septum length nor implantation depth > membranous septum depth to be predictive of major conduction disturbance, although CIs were wide and the possibility of a type II statistical error cannot be excluded.

One factor that was not predictive of conduction disturbance was calcium volume. We examined a number of calcium features that have previously been found to be predictive of PPM in patients implanted with balloon-expandable THVs but did not confirm these findings.^16^ This study examined self-expanding devices, and differences in device technology might explain this discrepancy in outcomes.

In this study, the computer simulations did not demonstrate perfect diagnostic accuracy. It is important to recognize that the computer modeling identifies a region of interest inferior to the membranous septum, with the assumption that this area represents the atrioventricular bundle and proximal left bundle branch. The membranous septum is only an anatomical surrogate for the atrioventricular bundle, and a detailed anatomical analysis has demonstrated significant heterogeneity in the location of the atrioventricular bundle.^31^ While the atrioventricular bundle most commonly passes along the lower border of the membranous septum, the bundle may also pass within the membranous septum or through the muscular septum. This anatomical variation may, in part, explain the somewhat modest diagnostic accuracy of the computer simulations. Furthermore, the optimal CPMax cutoff demonstrated poor sensitivity, potentially limiting the clinical utility of this conduction disturbance modeling parameter.

We demonstrated that patients who required PPM implantation had a longer postprocedural length of stay. These findings are consistent with those of previous studies which had demonstrated that PPM implantation was associated with both a longer ICU and hospital length of stay.^25^^,^^26^ Furthermore, a high contact pressure was associated with a longer postprocedural length of stay. Since CPMax was a predictor for PPM, a plausible mechanism is provided for the ability of the computer simulations to predict postprocedural length of stay.

We identified that patients who developed major conduction disturbance did not have any significant change in LVEF. These findings are consistent with prior work, demonstrating a relationship between LVEF and LBBB.^32^ CPMax was predictive for a change in LVEF, but CPI was not predictive for this clinical outcome. CIs were broad, and this finding may potentially represent a type 2 statistical error. We also demonstrated that LVEF did not improve in patients requiring PPM implantation, again, consistent with prior findings.^33^

In this study, patients who required a PPM for third-degree atrioventricular block had a higher risk of long-term mortality, as has previously been demonstrated.^34^ The predicted CPMax from the computer simulations was also found to identify a group of patients at a higher risk for death. Since CPMax was found to be a risk factor for major conduction disturbance, PPM implantation, and reduction in LVEF, a potential mechanism is provided for the ability of the computer simulations to predict long-term mortality. Furthermore, it might be hypothesized that an elevated CPMax could potentially be predictive of late heart block or sudden cardiac death. However, CIs were broad, and the possibility of type I statistical error cannot be excluded. Furthermore, cause of death information was not available. These findings should be considered exploratory, and further validation will be required in larger, sufficiently powered studies.

In this study, patient-specific computer simulation was performed using FEops HEARTguide technology. A number of alternate patient-specific computer models have been developed demonstrating that parameters such as maximal principal strain^9^ and von Mises stresses may predict conduction disturbance.^7^

This study included only a limited number of patients with bicuspid aortic valve. This anatomy has been associated with a shorter membranous septum length,^35^ which places these patients at a higher risk for conduction abnormalities.^30^ While the membranous septum length is incorporated into the modeling process, further validation in this important patient subgroup is needed.

Previous studies have identified that the 34-mm Evolut prosthesis is a risk factor for conduction disturbance,^36^ and while this finding was not seen in this study, we found that CPMax was higher with larger prosthesis sizes, providing a plausible mechanism for the increased incidence of conduction disturbance seen with the 34-mm Evolut R THV in prior work.

**Figure 6:**
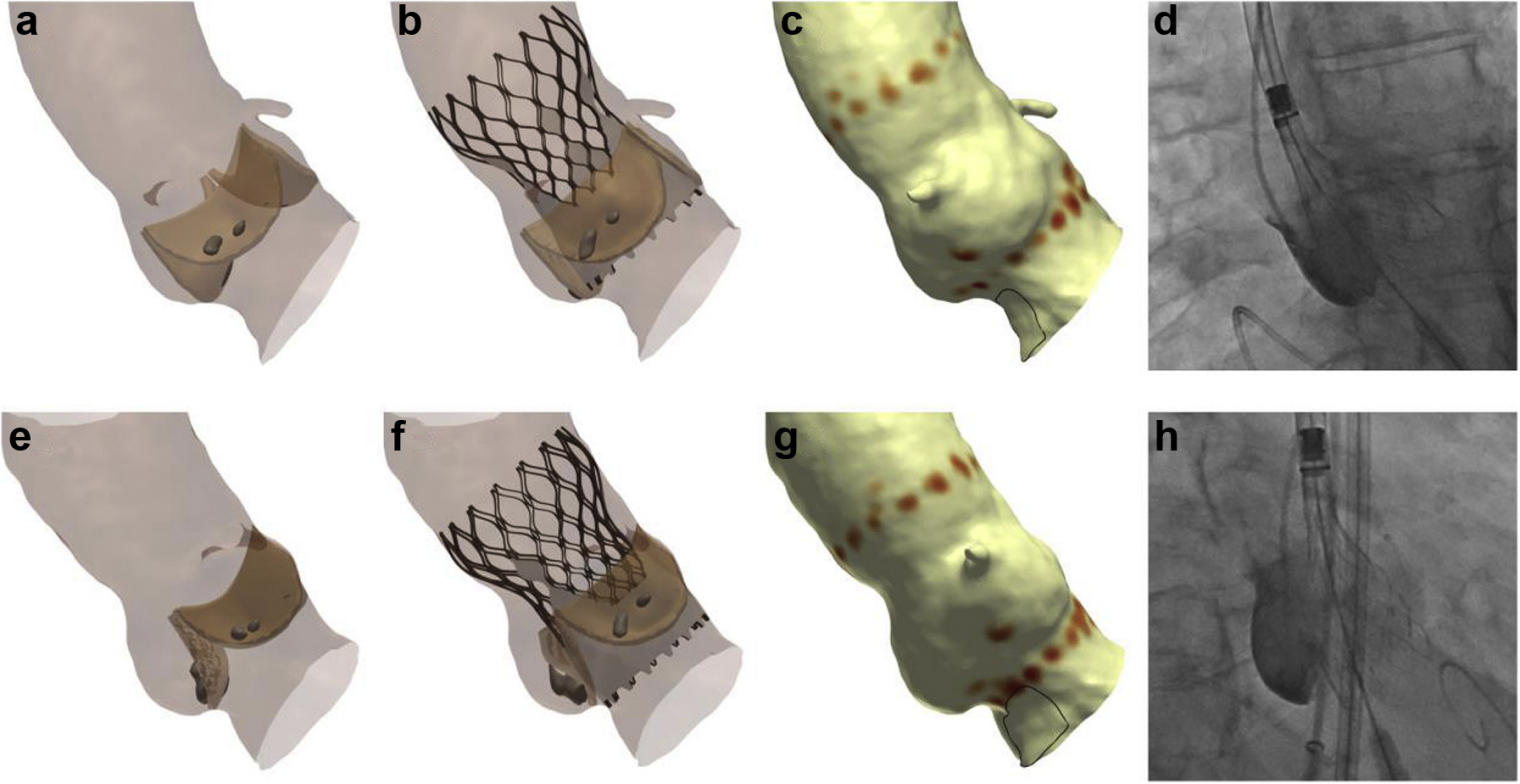
**Potential utility for patient-specific transcatheter heart valve deployment.** (a) A finite element model of the aortic root has been created, and a 3-cusp view identified. (b) Finite element analysis is performed to simulate the implanted THV. (c) Conduction disturbance modeling indicates a low risk of conduction disturbance (CPI 4%, CPMax 0.38 MPa). (d) During the procedure, the computer simulations could be used to match this target implant depth. (e-h) This process can be repeated in the cusp-overlay view. Abbreviations: CPI, contact pressure index; CPMax, maximum contact pressure; THV, transcatheter heart valve.

Moving forward, it is important to consider how a technology such as patient-specific computer simulation might be incorporated into clinical practice. In this study, we demonstrated that additional computer modeling could be performed to identify a THV implantation depth that would minimize conduction disturbance in the vast majority of patients. We would suggest that for patients undergoing transcatheter aortic valve implantation, this technology could potentially be used to identify an optimal implantation depth to minimize conduction disturbance.^37^ Computer simulation could then be used to guide the procedure in both the 3-cusp and cusp-overlay views (Figure 6).^38^^,^^39^ Imaging fusion technology could also potentially be used to guide valve deployment.^40^

**Figure 7:**
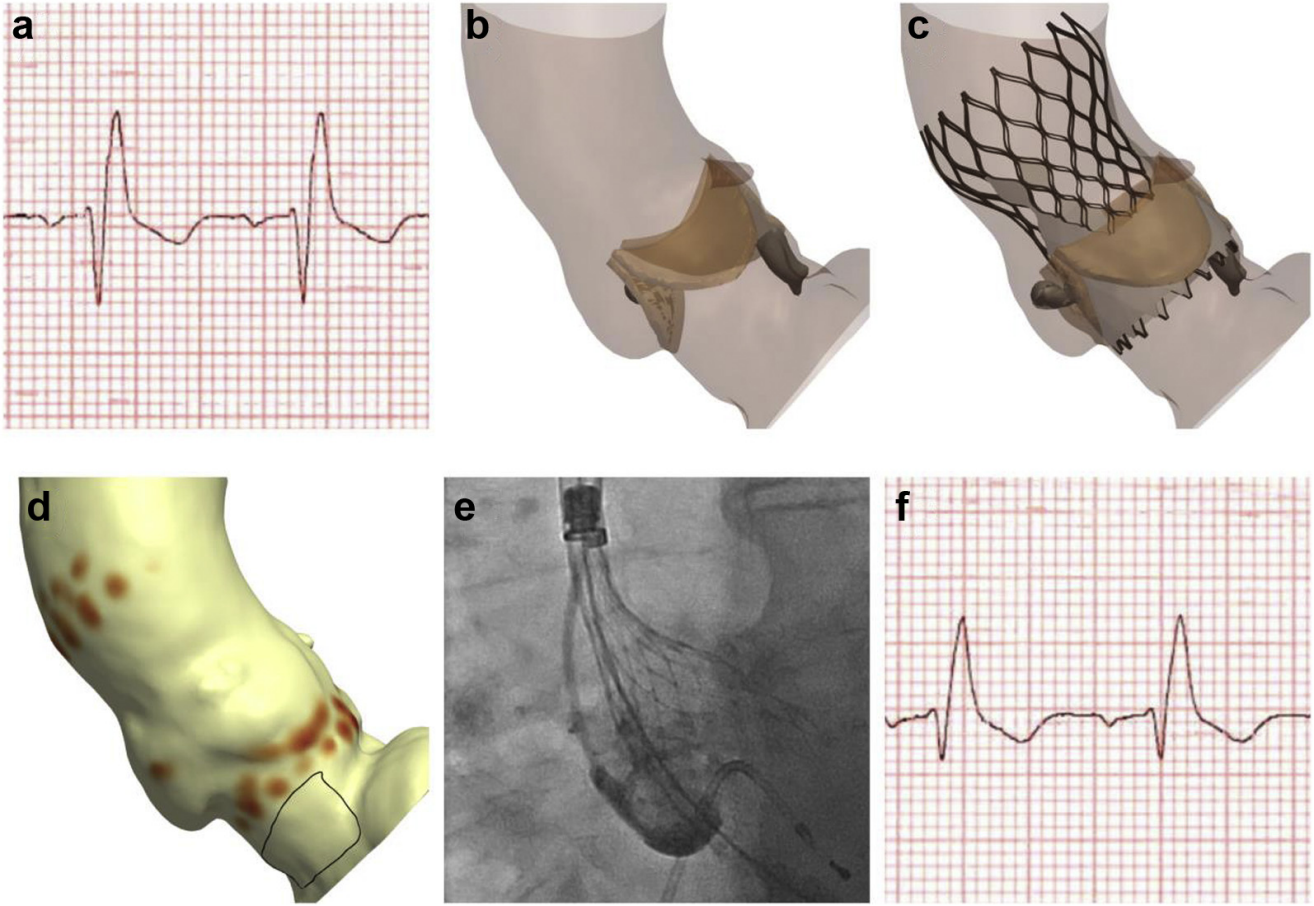
**Patient-specific computer simulation in a patient at high risk for conduction disturbance.** (a) A patient with pre-existing RBBB (b) had a finite element model of their aortic root created. (c) Finite element analysis and (d) conduction disturbance modeling is performed, demonstrating a low risk of conduction disturbance (CPI 1%, CPMax 0.05 MPa). (e) Deployment of a THV at this implantation depth (f) did not result in any new conduction disturbance. Abbreviations: CPI, contact pressure index; CPMax, maximum contact pressure; RBBB, right bundle branch block; THV, transcatheter heart valve.

Ideally, all patients would be considered for patient-specific computer simulation, as the usage of this technology has been demonstrated to alter procedural elements, such as target depth of implantation.^41^ However, due to time and financial constraints, this technology might selectively be used for patients at high risk for conduction disturbance, such as those with pre-existing right bundle branch block (Figure 7), for patients with reduced left ventricular ejections, where permanent pacing may have deleterious long-term outcomes, or for patients with complex anatomy, such as bicuspid aortic valve.^37^^,^^42^, ^43^, ^44^ Furthermore, patient-specific computer simulation is just one of a number of potential precision medicine techniques that may be incorporated into transcatheter aortic valve implantation procedural planning, execution, and follow-up. Components of such an approach might include identification of membranous septum height,^36^ deep learning models,^45^ and the usage of rapid atrial pacing.^46^

### Limitations

It is important to recognize the significant limitations of this study. First, this was a small, retrospective study, involving a limited number of centers, and further validation of the role of this technology is required in larger prospective studies. A significant number of patients could not undergo conduction disturbance modeling due to inadequate right-sided contrast opacification. The frequency of conduction disturbance in this study was high, as our study predates the widespread usage of the cusp-overlay technique,^38^ which has been demonstrated to reduce the incidence of LBBB and PPM implantation, when compared to deployment in the 3-cusp coplanar view.^39^ Implantation depth was assessed using procedural contrast angiography, using a nonstandardized fluoroscopic projection, which may have introduced the possibility of parallax errors,^47^ and our study would be strengthened through the usage of postprocedural cardiac CT imaging to more accurately gauge THV implantation depth. Achieving target implantation depth may be challenging with self-expanding technology as device microdislodgement is frequent with these devices.^48^ Furthermore, device positioning may be challenging in horizontal aortas, and this anatomical feature is associated with a higher incidence of LBBB and PPM.^49^ Even though hyperelastic models may better represent the mechanical behavior of the aortic root tissue, elastic material properties were used to model the aortic root and valve tissues. However, this linearization seems viable as previous studies have reported that the accuracy of the predicted THV frame deformation using a simple linear elastic material or a more complex hyperelastic material is comparable.^50^^,^^51^ In this study, frame rotation was not accounted for, although this factor has previously been demonstrated to not significantly influence the conduction disturbance modeling.^8^ While the computer models account for predilation,^8^ repositioning and postdilatation are not modeled, and both of these factors are known to be associated with conduction disturbance.^52^^,^^53^ The utility of the computer simulations might be mitigated by a routine strategy targeting a high implantation depth; however, a high implantation may lead to THV embolization, a complication which is associated with a number of adverse clinical outcomes, including all-cause mortality and stroke.^54^ Although validation of the computer models has previously been undertaken, demonstrating accurate prediction of the THV frame morphology and calcium displacement,^17^ no validation has been performed comparing differences in aortic root dimensions between CT-derived measurements and the finite element models, potentially limiting the accuracy of the conduction disturbance modeling. The simulations were performed at a static phase of the cardiac cycle; therefore, the possible influence of cardiac motion on the contact-related parameters were not accounted for in this study and further investigation should be performed to evaluate whether heart motion and surrounding tissues (e.g., left atrium) influence the contact pressure measurements. Postprocedural length of stay is influenced by both patient comorbidities and procedural complications,^55^ which were not accounted for in this study. Finally, this study examined self-expanding devices and further validation of the technology with balloon-expandable THVs is required.

## Conclusion

Patient-specific computer simulation may be used to identify patients at risk for conduction disturbance after TAVR with current-generation self-expanding THVs. This technology could potentially be used to plan and guide procedural aspects to minimize the risk of conduction disturbance and its associated adverse clinical outcomes.

## Ethics Statement

The study protocol was approved by a local human research ethics committee (Monash Health Human Research Ethics Committee RES-21-0000-379L). Informed consent was not required due to the retrospective and low-risk nature of the research.

## Funding

This study is part of a project that has received funding from the 10.13039/100010661European Union’s Horizon 2020 research and innovation program under grant agreement No. 945698. Dr Hashrul N. Rashid is supported by the Postgraduate Scholarship (Reference No. APP 1191131) from the National Health and Medical Research Council (NHMRC) (Sydney, NSW, Australia), Health Professional Scholarship (Reference No. 102452) from the National Heart Foundation of Australia (Sydney, NSW, Australia) and the Kincaid-Smith Scholarship from the Royal Australasian College of Physicians (RACP) (Sydney, NSW, Australia).

## Disclosure Statement

Dr Dowling reports grants from 10.13039/100004374Medtronic, outside the submitted work. A/Prof. Gooley reports personal fees from Boston Scientific, outside the submitted work. Dr McCormick reports personal fees from Boston Scientific, outside the submitted work. Prof. Brecker reports grants and personal fees from 10.13039/100004374Medtronic, outside the submitted work. All other authors have nothing to disclose.

## References

[1] Dowling C., Kondapally Seshasai S.R., Firoozi S., Brecker S.J. (2020). Transcatheter aortic valve replacement versus surgery for symptomatic severe aortic stenosis: a reconstructed individual patient data meta-analysis. Catheter Cardiovasc Interv.

[2] Carroll J.D., Mack M.J., Vemulapalli S. (2020). STS-ACC TVT Registry of transcatheter aortic valve replacement. J Am Coll Cardiol.

[3] Mack M.J., Leon M.B., Thourani V.H. (2019). Transcatheter aortic-valve replacement with a balloon-expandable valve in low-risk patients. N Engl J Med.

[4] Thyregod H.G., Steinbrüchel D.A., Ihlemann N. (2015). Transcatheter versus surgical aortic valve replacement in patients with severe aortic valve stenosis: 1-year results from the all-comers NOTION randomized clinical trial. J Am Coll Cardiol.

[5] Popma J.J., Deeb G.M., Yakubov S.J. (2019). Transcatheter aortic-valve replacement with a self-expanding valve in low-risk patients. N Engl J Med.

[6] Jørgensen T.H., De Backer O., Gerds T.A., Bieliauskas G., Svendsen J.H., Søndergaard L. (2019). Mortality and heart failure hospitalization in patients with conduction abnormalities after transcatheter aortic valve replacement. JACC Cardiovasc Interv.

[7] Bianchi M., Marom G., Ghosh R.P. (2016). Effect of balloon-expandable transcatheter aortic valve replacement positioning: a patient-specific numerical model. Artif Organs.

[8] Rocatello G., El Faquir N., De Santis G. (2018). Patient-specific computer simulation to elucidate the role of contact pressure in the development of new conduction abnormalities after catheter-based implantation of a self-expanding aortic valve. Circ Cardiovasc Interv.

[9] Bosi G.M., Capelli C., Cheang M.H. (2020). A validated computational framework to predict outcomes in TAVI. Sci Rep.

[10] Dowling C., Firoozi S., Doyle N. (2019). Initial experience of a large, self-expanding, and fully recapturable transcatheter aortic valve: the UK & Ireland Implanters' Registry. Catheter Cardiovasc Interv.

[11] Dowling C., Firoozi S., Panoulas V. (2020). Initial experience of a self-expanding transcatheter aortic valve with an outer pericardial wrap: the United Kingdom and Ireland Implanters' Registry. Catheter Cardiovasc Interv.

[12] Sievers H.H., Schmidtke C. (2007). A classification system for the bicuspid aortic valve from 304 surgical specimens. J Thorac Cardiovasc Surg.

[13] Jilaihawi H., Chen M., Webb J. (2016). A bicuspid aortic valve imaging classification for the TAVR era. JACC Cardiovasc Imaging.

[14] Buellesfeld L., Stortecky S., Kalesan B. (2013). Aortic root dimensions among patients with severe aortic stenosis undergoing transcatheter aortic valve replacement. JACC Cardiovasc Interv.

[15] Khalique O.K., Hahn R.T., Gada H. (2014). Quantity and location of aortic valve complex calcification predicts severity and location of paravalvular regurgitation and frequency of post-dilation after balloon-expandable transcatheter aortic valve replacement. JACC Cardiovasc Interv.

[16] Maeno Y., Abramowitz Y., Kawamori H. (2017). A highly predictive risk model for pacemaker implantation after TAVR. JACC Cardiovasc Imaging.

[17] Schultz C., Rodriguez-Olivares R., Bosmans J. (2016). Patient-specific image-based computer simulation for theprediction of valve morphology and calcium displacement after TAVI with the Medtronic CoreValve and the Edwards SAPIEN valve. EuroIntervention.

[18] Massing G.K., James T.N. (1976). Anatomical configuration of the His bundle and bundle branches in the human heart. Circulation.

[19] Urena M., Webb J.G., Cheema A. (2014). Impact of new-onset persistent left bundle branch block on late clinical outcomes in patients undergoing transcatheter aortic valve implantation with a balloon-expandable valve. JACC Cardiovasc Interv.

[20] Houthuizen P., Van Garsse L.A., Poels T.T. (2012). Left bundle-branch block induced by transcatheter aortic valve implantation increases risk of death. Circulation.

[21] Urena M., Webb J.G., Eltchaninoff H. (2015). Late cardiac death in patients undergoing transcatheter aortic valve replacement: incidence and predictors of advanced heart failure and sudden cardiac death. J Am Coll Cardiol.

[22] Nazif T.M., Williams M.R., Hahn R.T. (2014). Clinical implications of new-onset left bundle branch block after transcatheter aortic valve replacement: analysis of the PARTNER experience. Eur Heart J.

[23] Testa L., Latib A., De Marco F. (2013). Clinical impact of persistent left bundle-branch block after transcatheter aortic valve implantation with CoreValve revalving system. Circulation.

[24] Chamandi C., Barbanti M., Munoz-Garcia A. (2019). Long-term outcomes in patients with new-onset persistent left bundle branch block following TAVR. JACC Cardiovasc Interv.

[25] Fadahunsi O.O., Olowoyeye A., Ukaigwe A. (2016). Incidence, predictors, and outcomes of permanent pacemaker implantation following transcatheter aortic valve replacement: analysis from the U.S. Society of Thoracic Surgeons/American College of Cardiology TVT Registry. JACC Cardiovasc Interv.

[26] Nazif T.M., Dizon J.M., Hahn R.T. (2015). Predictors and clinical outcomes of permanent pacemaker implantation after transcatheter aortic valve replacement: the PARTNER (Placement of AoRtic TraNscathetER Valves) trial and registry. JACC Cardiovasc Interv.

[27] Buellesfeld L., Stortecky S., Heg D. (2012). Impact of permanent pacemaker implantation on clinical outcome among patients undergoing transcatheter aortic valve implantation. J Am Coll Cardiol.

[28] Kalogeras K., Ruparelia N., Kabir T. (2020). Comparison of the self-expanding Evolut-PRO transcatheter aortic valve to its predecessor Evolut-R in the real world multicenter ATLAS registry. Int J Cardiol.

[29] Ojeda S., Hidalgo F., Romero M. (2020). Impact of the repositionable Evolut R CoreValve system on the need for a permanent pacemaker after transcatheter aortic valve implantation in patients with severe aortic stenosis. Catheter Cardiovasc Interv.

[30] Hamdan A., Guetta V., Klempfner R. (2015). Inverse relationship between membranous septal length and the risk of atrioventricular block in patients undergoing transcatheter aortic valve implantation. JACC Cardiovasc Interv.

[31] Kawashima T., Sasaki H. (2005). A macroscopic anatomical investigation of atrioventricular bundle locational variation relative to the membranous part of the ventricular septum in elderly human hearts. Surg Radiol Anat.

[32] Nazif T.M., Chen S., George I. (2019). New-onset left bundle branch block after transcatheter aortic valve replacement is associated with adverse long-term clinical outcomes in intermediate-risk patients: an analysis from the PARTNER II trial. Eur Heart J.

[33] Urena M., Webb J.G., Tamburino C. (2014). Permanent pacemaker implantation after transcatheter aortic valve implantation: impact on late clinical outcomes and left ventricular function. Circulation.

[34] Sammour Y., Krishnaswamy A., Kumar A. (2021). Incidence, predictors, and implications of permanent pacemaker requirement after transcatheter aortic valve replacement. JACC Cardiovasc Interv.

[35] Hamdan A., Nassar M., Schwammenthal E. (2021). Short membranous septum length in bicuspid aortic valve stenosis increases the risk of conduction disturbances. J Cardiovasc Comput Tomogr.

[36] Jilaihawi H., Zhao Z., Du R. (2019). Minimizing permanent pacemaker following repositionable self-expanding transcatheter aortic valve replacement. JACC Cardiovasc Interv.

[37] Dowling C., Gooley R., McCormick L., Firoozi S., Brecker S.J. (2021). Patient-specific computer simulation: an emerging technology for guiding the transcatheter treatment of patients with bicuspid aortic valve. Interv Cardiol.

[38] Tang G.H.L., Zaid S., Michev I. (2018). “Cusp-overlap” view simplifies fluoroscopy-guided implantation of self-expanding valve in transcatheter aortic valve replacement. JACC Cardiovasc Interv.

[39] Mendiz O.A., Noč M., Fava C.M. (2021). Impact of cusp-overlap view for TAVR with self-expandable valves on 30-day conduction disturbances. J Interv Cardiol.

[40] Brouwer J., Ten Berg J.M., Rensing B., Swaans M.J. (2019). First use of futuristic image fusion technology during transcatheter aortic valve replacement. JACC Cardiovasc Interv.

[41] El Faquir N., De Backer O., Bosmans J. (2020). Patient-specific computer simulation in TAVR with the self-expanding Evolut R valve. JACC Cardiovasc Interv.

[42] Dowling C., Bavo A.M., El Faquir N. (2019). Patient-specific computer simulation of transcatheter aortic valve replacement in bicuspid aortic valve morphology. Circ Cardiovasc Imaging.

[43] Dowling C., Firoozi S., Brecker S.J. (2020). First-in-human experience with patient-specific computer simulation of TAVR in bicuspid aortic valve morphology. JACC Cardiovasc Interv.

[44] Dowling C., Gooley R., McCormick L. (2021). Patient-specific computer simulation to optimize transcatheter heart valve sizing and positioning in bicuspid aortic valve. Struct Heart.

[45] Galli V., Loncaric F., Rocatello G. (2021). Towards patient-specific prediction of conduction abnormalities induced by transcatheter aortic valve implantation: a combined mechanistic modelling and machine learning approach. Eur Heart J Digital Health.

[46] Krishnaswamy A., Sammour Y., Mangieri A. (2020). The utility of rapid atrial pacing immediately post-TAVR to predict the need for pacemaker implantation. JACC Cardiovasc Interv.

[47] Thériault-Lauzier P., Andalib A., Martucci G. (2014). Fluoroscopic anatomy of left-sided heart structures for transcatheter interventions: insight from multislice computed tomography. JACC Cardiovasc Interv.

[48] Hellhammer K., Piayda K., Afzal S. (2019). Micro-dislodgement during transcatheter aortic valve implantation with a contemporary self-expandable prosthesis. PLoS One.

[49] Gallo F., Gallone G., Kim W.K. (2021). Horizontal aorta in transcatheter self-expanding valves: insights from the HORSE International Multicentre Registry. Circ Cardiovasc Interv.

[50] Finotello A., Morganti S., Auricchio F. (2017). Finite element analysis of TAVI: impact of native aortic root computational modeling strategies on simulation outcomes. Med Eng Phys.

[51] Russ C., Hopf R., Hirsch S. (2013). Simulation of transcatheter aortic valve implantation under consideration of leaflet calcification. Annu Int Conf IEEE Eng Med Biol Soc.

[52] Rashid H.N.Z., Gooley R., McCormick L. (2017). Safety and efficacy of valve repositioning during transcatheter aortic valve replacement with the Lotus Valve System. J Cardiol.

[53] Chen S., Chau K.H., Nazif T.M. (2020). The incidence and impact of cardiac conduction disturbances after transcatheter aortic valve replacement. Ann Cardiothorac Surg.

[54] Kim W.K., Schäfer U., Tchetche D. (2019). Incidence and outcome of peri-procedural transcatheter heart valve embolization and migration: the TRAVEL registry (TranscatheteR HeArt Valve EmboLization and Migration). Eur Heart J.

[55] Durand E., Avinée G., Gillibert A. (2021). Analysis of length of stay after transfemoral transcatheter aortic valve replacement: results from the FRANCE TAVI registry. Clin Res Cardiol.

