# Supplementary material for: Patient-Specific Computer Simulation to Predict Conduction Disturbance With Current-Generation Self-Expanding Transcatheter Heart Valves
Source: Struct Heart. 2022 Mar 29;6(3):100010. doi: 10.1016/j.shj.2022.100010 (PMC10236875; doi:10.1016/j.shj.2022.100010)
Supplement: Supplemental Table 1 [file mmc1.docx]

Supplementary Table 1. Procedural Characteristics.

| Characteristic | n = 80 |
| --- | --- |
| Local anaesthesia with sedation | 65 (81.3) |
| Pre-dilation | 39 (48.8) |
| Transcatheter heart valve |  |
| 23 mm Evolut PRO | 1 (1.3) |
| 26 mm Evolut PRO | 17 (21.3) |
| 29 mm Evolut PRO | 35 (43.8) |
| 34 mm Evolut R | 27 (33.8) |
| THV implantation depth | 6.2 ± 2.3 |
| Non-coronary cusp | 5.5 ± 2.6 |
| Left coronary cusp | 6.9 ± 2.4 |
| Post-dilation | 8 (10.0) |

THV denotes transcatheter heart valve.
